# A Novel Multiplex LAMP Assay for the Detection of Respiratory Human Adenoviruses

**DOI:** 10.3390/ijms25137215

**Published:** 2024-06-29

**Authors:** Maksim A. Koryukov, Igor P. Oscorbin, Lidiya M. Novikova, Maria A. Gordukova, Irina E. Turina, Elena V. Galeeva, Dmitry A. Kudlay, Maxim L. Filipenko

**Affiliations:** 1 The Institute of Chemical Biology and Fundamental Medicine, Siberian Branch of the Russian Academy of Sciences, 8 Lavrentiev Avenue, Novosibirsk 630090, Russia; mkoryukov@gmail.com (M.A.K.);; 2 Department of Natural Sciences, Novosibirsk State University, Novosibirsk 630090, Russia; 3 G. Speransky Children’s Hospital No. 9, 29 Shmitovsky Prospect, Moscow 123317, Russia; 4 Department of Natural Sciences, I.M. Sechenov First Moscow State Medical University, Pogodinskaya St. 1, Moscow 119991, Russia

**Keywords:** multiplex, LAMP, adenoviruses, HAdV, PCR, qPCR, melting curves, respiratory infections

## Abstract

Human adenoviruses (HAdVs) are common pathogens that are associated with a variety of diseases, including respiratory tract infections (RTIs). Without reliable, fast, and cost-effective detection methods for HAdVs, patients may be misdiagnosed and inappropriately treated. To address this problem, we have developed a multiplex loop-mediated isothermal amplification (LAMP) assay for the detection of the species *Human adenovirus B* (HAdV-B), *Human adenovirus C* (HAdV-C) and *Human adenovirus E* (HAdV-E) that cause RTIs. This multiplexing approach is based on the melting curve analysis of the amplicons with a specific melting temperature for each HAdV species. Without the need for typing of HAdVs, the LAMP results can be visually detected using colorimetric analysis. The assay reliably detects at least 375 copies of HAdV-B and -C and 750 copies of HAdV-E DNA per reaction in less than 35 min at 60 °C. The designed primers have no in silico cross-reactivity with other human respiratory pathogens. Validation on 331 nasal swab samples taken from patients with RTIs showed a 90–94% agreement rate with our in-house multiplex quantitative polymerase chain reaction (qPCR) method. Concordance between the quantitative and visual LAMP was 99%. The novel multiplexed LAMP could be an alternative to PCR for diagnostic purposes, saving personnel and equipment time, or could be used for point-of-care testing.

## 1. Introduction

Respiratory tract infections, most notably lower respiratory infections (LRIs), are among the main causes of illness and death in the world. In 2019, globally, there were nearly 490 million cases of RTIs and 2.4 million deaths from LRIs [1]. According to the World Health Organization (WHO), RTIs rank among the five most impactful causes of the disease burden when measured using disability-adjusted life-years (DALYs, a time-based measure that represents years of life lost due to premature mortality and years of life lost due to time lived in states of less than full health, or due to disability) [2]. While this remains a serious challenge for healthcare providers in both developed and developing countries, the mortality and morbidity rates of RTIs significantly decreased in almost all regions between 1990 and 2019. The COVID-19 pandemic has demonstrated weaknesses in modern methods of regulating the airborne transmission of communicable diseases [3]. Several groups of respiratory viruses have been somewhat neglected in terms of diagnostic and clinical management. For example, the transmission of the year-round viruses, such as adenoviruses, is not regulated, and the real disease burden has yet to be determined [4].

Adenoviruses, which belong to the *Adenoviridae* family, can be found almost everywhere in nature and can infect a variety of tissues and organs, causing a wide range of symptoms. These viruses are highly resistant to almost any environment, and they remain infectious for up to three weeks [5]. According to current taxonomy, there are seven different species of adenovirus that can infect humans, and each one has a different tissue tropism. HAdV-C, -E and some -B serotypes infect the respiratory tract, and other HAdV-B serotypes replicate in the urinary tract; *Human adenovirus A* and *Human adenovirus F* species are associated with infections in the gastrointestinal tract, while *Human adenovirus D* species cause eye infections. This paper is focused only on adenoviruses that are associated with respiratory tract infections. Although most of the adenovirus infections are mild upper respiratory diseases, some can cause severe LRIs that can lead to death, even in relatively healthy individuals [6]. HAdV outbreaks have been reported in crowded areas, such as hospitals, military camps and schools all over the world [7,8,9,10]. Currently, there is no approved antiviral medication for treating HAdV infections. Several commercial antiviral drugs, such as ganciclovir, cidofovir and ribavirin, are effective in vitro against various HAdV types [11,12,13]. However, there has been no observed therapeutic effect in vivo at safe concentrations of these drugs. There is currently no vaccine available to the public that would prevent adenovirus transmission, making it impossible to completely stop viral spread. Therefore, the only ways to control this disease are through timely diagnostics and subsequent prevention through quarantine measures.

When it comes to diagnosing adenovirus infections, various types of samples can be used, including nasopharyngeal aspirates and swabs. These samples can then be analyzed using various methods, with polymerase chain reaction (PCR) being considered the “gold standard”. Numerous laboratory-developed and commercial PCR kits are available all over the world [14,15,16,17]. These kits allow for the detection of adenoviruses in various samples. Different multiplex PCR panels can target most, if not all, of the known infectious agents that can affect the human respiratory system [18,19,20,21]. PCR is also widely used for detecting HAdV infections [22,23]. While PCR is a relatively fast and accurate method, it does have several limitations. During the COVID-19 pandemic, the increased demand for diagnostic testing and the lack of sufficient equipment restricted the number of PCR tests that could be performed daily [24]. Other methods like virus isolation are not widely used due to being highly labor-intensive and time-consuming [25]. Enzyme-linked immunosorbent assay (ELISA) can also be used for the detection of viruses, but it has relatively lower sensitivity and specificity with a high rate of false positive results and a low signal-to-noise ratio. Next-generation sequencing (NGS) technologies are also being used to detect respiratory pathogens. However, current challenges, such as high costs and poor logistics, are preventing their widespread implementation in clinical practice. These challenges necessitate the development of new diagnostic tools and the refinement of existing ones in order to increase the throughput of clinical laboratories and improve patient outcomes.

During the last few decades, numerous alternative methods have been suggested as PCR alternatives, and many of these rely on isothermal amplification. Examples include Nucleic Acid Sequence-Based Amplification (NASBA) [26], Exponential Strand Displacement Amplification (E-SDA) [27], Hyperbranching Rolling Circle Amplification (HRCA) [28], Primer Generation–Rolling Circle Amplification (PG-RCA) [29], LAMP [30], Helicase-Dependent Amplification (HDA) [31], Recombinase Polymerase Amplification (RPA) [32], Exponential Amplification Reaction (EXPAR) [33], Multiple Displacement Amplification (MDA) [34], Primase-based Whole Genome (pWGA) [35] and other recently developed techniques [36]. Isothermal amplification allows for analysis without the need for expensive thermocycler equipment, making it almost ideal for point-of-care testing in remote areas. Among the isothermal amplification techniques, LAMP has emerged as a promising and popular method. LAMP relies on the use of DNA polymerases with strand displacement activity and the use of two or three primer pairs. The reaction products can be detected visually using colorimetric analysis [37], eliminating the need for complex optical detection systems. In terms of specificity and sensitivity, LAMP is generally identical to PCR [38], although it is approximately three times faster. Like PCR, LAMP can be used to simultaneously detect two or more target pathogens. However, it is a challenging task to multiplex LAMP primers due to the formation of non-specific products, such as primer dimers and hairpin structures. These byproducts significantly reduce the efficiency of the reaction, thus decreasing the analytical sensitivity and specificity of LAMP assays [39]. Nevertheless, several studies have described multiplex LAMP assays for the detection of SARS-CoV-2 [40] and other pathogens [41,42].

Here, we have developed a method for detecting HAdV types B, -C and -E DNA using multiplex loop-mediated isothermal amplification. The assay can be used to identify adenoviruses that are associated with human respiratory tract infections. This approach, coupled with melting curve analysis, allows for additional differentiation between different species of HAdVs. To validate our results, we compared our multiplex LAMP method with an in-house multiplex PCR panel on 331 clinical samples (nasal swabs) taken from patients with upper respiratory infections.

## 2. Results

### 2.1. Selection of LAMP Primers

Primers were designed based on the alignment of 82 HAdV-C, 533 HAdV-B and 77 HAdV-E complete genomes. Highly conserved sequences were identified using an in-house Python script, and consensus sequences served as templates for primer design. Preliminary estimations of the melting temperatures of the amplicons were not conducted in silico. Most of the primer sets from Primer Explorer v5 were filtered out due to failure to design LF/LB primers with specific melting temperatures. Primer sets were chosen based on their melting temperatures: 60 °C for LF/LB, F2/B2 and 64 °C for F1c/B1c. Out of all the sets selected, one for each type of HAdV was chosen for further experiments. Preliminary LAMP amplification results revealed efficient amplification even in sets with a high Gibbs free energy (data not provided). Therefore, we combined (multiplexed) primers for HAdV-C, -B and -E in the absence of in silico experiments due to a lack of understanding of the principles behind primer–dimer formation in LAMP reactions.

### 2.2. Optimization of Quantitative LAMP (qLAMP)

**Figure 1:**
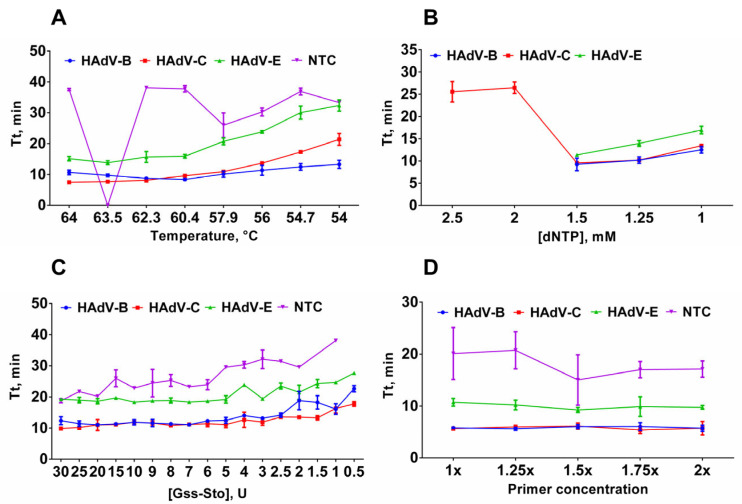
Optimization of multiplex LAMP conditions. (**A**) Optimal temperature for LAMP. Temperature of the LAMP reaction ranged from 54 to 64 °C. (**B**) Titration of dNTP concentration in LAMP. dNTP concentration was titrated in the range of 1–2.5 mM. (**C**) Titration of Gss-Sto polymerase in LAMP. Gss-Sto concentration was titrated in the range of 0.5–30 U. (**D**) Titration of primer concentration in LAMP. Primer concentration was titrated in the range of 1–2× (X× means that concentration of each primer in the triplex mixture was increased X times). Curves for each type are marked by a color: HAdV-B (blue/circle), -C (red/square), -E (green/triangle) and NTC (purple/inverted triangle); DNA template concentration was 2 × 10^3^ copies per reaction. Time-to-threshold (Tt) values are presented on the *Y*-axis, and the corresponding parameter (temperature, dNTP concentration, Gss-Sto concentration, primer concentration) is shown on the *X*-axis. Each experiment was triplicated, error bars represent one SD.

The simultaneous presence of 18 primers in the reaction significantly increased the chances of potential non-specific amplification, which led us to assume a relatively low sensitivity of the HAdV triplex prior to optimization. In order to increase the sensitivity of the assay, we optimized the qLAMP conditions, including concentrations of dNTP (1–2.5 mM), Gss-Sto polymerase (0.5–30 U), magnesium ions (4–16 mM), primers (from 1× to 2.5×); reaction temperature (52–62 °C) and time (25–40 min) (Figure 1).

**Figure 2:**
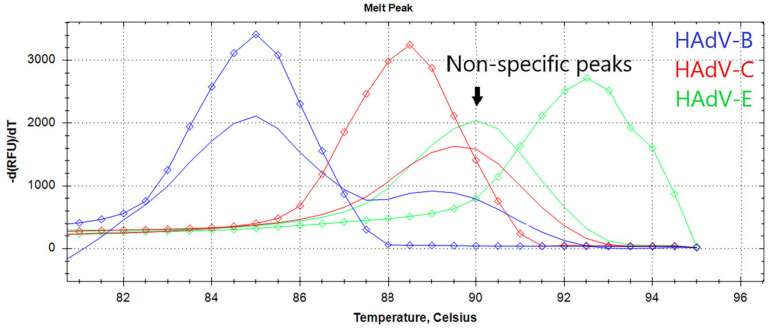
The melting curve analysis of LAMP products. The graph represents characteristic melting peaks for LAMP products obtained with the HAdV triplex. Curves for each type are marked by a color: HAdV-B (blue), -C (red), -E (green), diamonds indicate melting curves of positive control samples for each of the HAdV type. Non-specific melting peaks are in the range of 89–90.5 °C.

These particular parameters are believed to affect the sensitivity and reproducibility of LAMP assays. Plasmid controls were used as a control sample for LAMP optimization experiments. The standard LAMP conditions were as follows: 62 °C reaction temperature, 40 min reaction time, 1.6 μM FIP/BIP, 0.8 μM LF/LB, 0.4 μM F3/B3 primer concentrations, 3U of Gss-Sto polymerase, 2 × 10^4^ copies of DNA template per reaction. In each experiment, only one parameter was altered. To validate the LAMP product, melting curve analysis and polyacrylamide gel electrophoresis were conducted after each LAMP run. The estimated melting temperatures were approximately 84.5 °C for HAdV-B, 88 °C for HAdV-C, and 92 °C for HAdV-E amplicons (Figure 2). This difference is sufficient for HAdV typing in experiments with perfectly matched DNA templates.

Characteristic ladder-like patterns of LAMP products for each HadV type were assessed with polyacrylamide gel electrophoresis (Figures S1 and S2).

First, we determined the optimal temperature for LAMP. A high reaction temperature can enhance polymerase inactivation, while a low temperature facilitates the formation of reaction byproducts. The temperature range for LAMP was 54–64 °C. Based on the lowest observed Tt value, the optimal LAMP temperature was between 60 °C and 64 °C: 62 °C for HAdV-B, 60 °C for HAdV-C, 63.5 °C for HAdV-E (Figure 1A). Virtually, there was no difference in Tt values between these temperatures, so we assumed that the median temperature, i.e., 62 °C, is the optimal temperature for the HAdV LAMP multiplex test.

Next, we titrated the dNTPs in LAMP. A high dNTP concentration decreases the fidelity of the DNA polymerases and causes misincorporation during the synthesis, potentially leading to false-positive signals. Conversely, a deficiency in dNTP results in a lower amplification rate, which can lead to false-negative results, especially in samples with a low viral load. Here, the concentration range of dNTP was 1–2.5 mM, based on the commonly used concentration of 1.5 mM. An increase in the dNTP concentration to 2.5 and 2 mM completely inhibits LAMP (HAdV-B and HAdV-E), while a decrease to 1.25 and 1 mM inhibits LAMP partially (Figure 1B). Therefore, a concentration of 1.5 mM was selected as optimal.

After dNTPs, we optimized the concentration of Gss-Sto in LAMP. Similar to dNTPs, the concentration of DNA polymerase also needs to be balanced to achieve the best performance. This reduces the probability of false results during testing. The concentration of Gss-Sto polymerase was titrated between 0.5 and 30 units per reaction. Concentrations higher than 5U led to non-specific amplification in no template control (NTC) samples. Lowering the polymerase concentration below 2.5 U reduced reaction efficiency. Therefore, 3 U of Gss-Sto per reaction was used for further experiments.

After optimizing DNA polymerase concentrations, we varied primer concentrations within the range of 1–2 times the standard concentration. Increasing the primer concentration did not significantly improve the amplification efficiency. In fact, using more than 1.25× the primers resulted in significantly higher Tt values in the NTC (Figure 1D). In particular, increasing the HAdV-E primer concentration relative to other primers in the triplex resulted in efficient non-specific amplification in the NTCs (Figure S2). Therefore, primer concentration for each HAdV type remained 1×:1×:1× in the final reaction setup. Therefore, the final reaction setup maintained a 1:1:1 ratio for each HAdV species.

**Figure 3:**
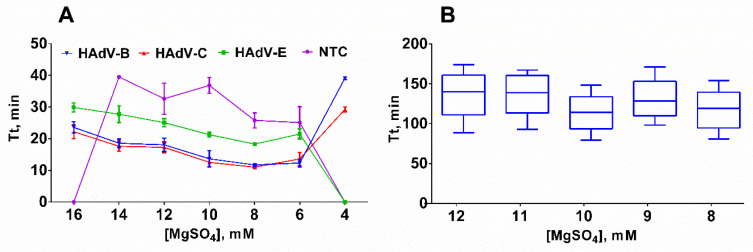
Titration of MgSO_4_ in LAMP. (**A**) Titration with a template; (**B**) titration without a template. Curves for each type are marked by a color: HAdV-B (blue/inverted triangle), -C (red/triangle), -E (green/square) and NTC (purple/circle). MgSO_4_ concentration was titrated in the range of 4–16 mM; DNA template concentration was 2 × 10^3^ copies per reaction. Time-to-threshold (Tt) values are presented on the *Y*-axis, the MgSO_4_ concentration is shown on the *X*-axis. Each experiment with a DNA template was triplicated and without a DNA template was replicated 10 times; error bars represent one SD.

Finally, we determined the optimal magnesium concentration by using serial dilutions of MgSO_4_ within the range of 4–16 mM. We observed the most efficient amplification with concentrations of 6–10 mM Mg^2+^, as concentrations lower than 6 mM almost completely inhibited the amplification, while concentrations higher than 10 mM only led to partial inhibition of LAMP (Figure 3A).

However, 3 technical replicates may not be sufficient for an accurate assessment of the LAMP reaction due to the high dispersion of Tt values observed in NTC samples. In order to validate our results, we titrated MgSO_4_ in the range of 8–12 mM in 10 technical replicates at each Mg^2+^ concentration. The results indicated that there was virtually no difference in the Tt values between 8 and 10 mM of Mg^2+^. Therefore, 8 mM of MgSO_4_ has been selected for further experiments based on the lowest Tt values obtained for positive control samples (Figure 3B).

### 2.3. Evaluation of qLAMP and Visual LAMP (vLAMP) ASSAYS Limits of Detection

The limit of detection (LoD) is a critical analytical parameter that defines the amount of analyte that can be detected in an assay. We evaluated the LoD of the designed LAMP assay by titrating the corresponding HAdV DNA template within the range of 50 to 1000 copies per reaction. For each concentration, experiments were conducted in 31 technical replicates in a single experiment. Replicates were defined as “positive” based on the presence of specific peaks on the melting curve (Figure 1). The LoD was defined as the DNA concentration at which specific melting peaks were observed in more than 95% of the technical repeats. In some cases, the difference between true negative and true positive replicates was less than 4 min, making it difficult to test based on the Tt value alone. Therefore, melting curve analysis is helpful for precisely calculating the LoD and testing samples with a low HAdV burden. False-positive samples can easily be identified by the presence of non-specific melting peaks at 89–90.5 °C on the melting curve (Figure 1).

**Figure 4:**
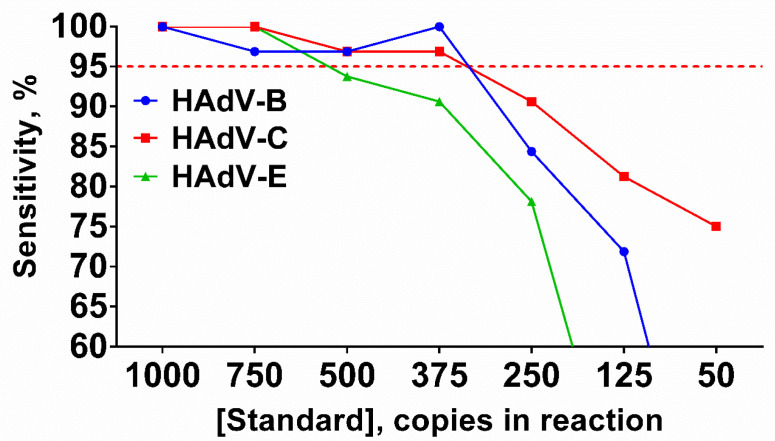
Sensitivity of the LAMP HAdV triplex. Curves for each type are marked by a color: HAdV-B (blue/circle), -C (red/square), -E (green/triangle). Red dotted line represents threshold line for LoD estimation. DNA templates were titrated in the range of 50–1000 copies per reaction. Sensitivity (%) values are presented on the *Y*-axis, DNA template concentration (copies per reaction) is shown on the *X*-axis. Each experiment was repeated 31 times for each DNA template concentration.

Sensitivity was calculated for each HAdV type and template concentration individually using the following equation: Sensitivity = Npositive/Ntotal × 100, where Npositive is the number of technical repeats with the relevant melting peaks for a given template concentration, Ntotal is the total number of technical repeats for a given template concentration. The sensitivity of the HAdV triplex significantly decreased when the DNA concentration was lower than 375 molecules per reaction. LoDs for HAdV-B and -C were 375 copies per reaction, whereas the LoD for HAdV-E was higher at 750 copies per reaction (Figure 4).

Identical experiments were conducted for vLAMP. Results for vLAMP are congruent with qLAMP.

### 2.4. Optimization of the LAMP Reaction Time

**Figure 5:**
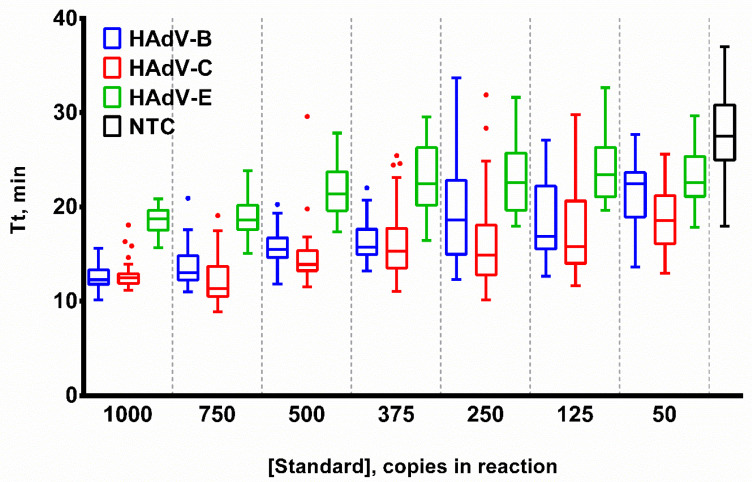
Template titration in LAMP. Curves for each HAdV type are marked by a color: HAdV-B (blue), -C (red), -E (green) and NTC (black). DNA template concentration was titrated in the range of 50–1000 copies per reaction. Time-to-threshold (Tt) values are presented on the *Y*-axis, DNA template concentrations are shown on the *X*-axis. Each experiment was repeated 31 times, error bars represent one SD, and dots represent outliers.

The duration of the vLAMP test was the most critical parameter to optimize, as the reaction time is strictly limited by the formation of byproducts in NTC samples. As a result, an excessive duration of the LAMP test would lead to false-negative results. However, the qLAMP results (Tt values) cannot be directly applied to the vLAMP test due to the end-point detection of the latter. Based on the number of positive NTC replicates, we suggest that the optimal time for the qLAMP test is 25–40 min (Figure 5).

**Figure 6:**
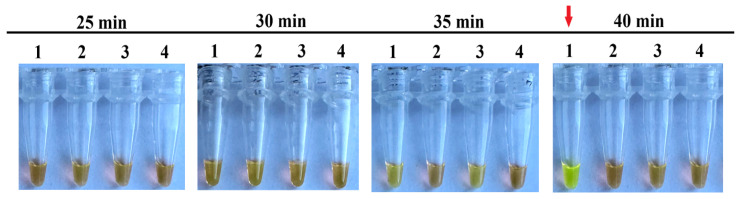
Optimal duration of vLAMP reaction. With each time, 4 technical replicates of vLAMP were performed without a DNA template. A color changed from orange (negative) to green (positive) in the first repeat at 40 min (marked by red arrow), indicating a false positive reaction.

We tested the duration of the vLAMP from 25 to 40 min at 60 °C in 4 NTC replicates. A slight green tint can be seen in the reactions after 35 min. However, it is easy to distinguish from the true positive samples. A clear color change from orange to green was observed in one replicate at 40 min (Figure 6). Thus, the duration for all further vLAMP reactions was 35 min.

### 2.5. Evaluation of qPCR Assay Limit of Detection

Preliminary experiments indicated that qPCR was more sensitive than the LAMP HAdV triplex. Therefore, we evaluated the LoD of the triplex PCR using a significantly lower amount of DNA template, ranging from 2 to 20 copies per reaction. For each concentration, experiments were conducted in 31 technical repeats in a single run, with an additional 48 repetitions for the lowest concentration (2 copies per reaction) for each HAdV type. It should be noted that we did not assess the LoD for HAdV-E due to the absence of clinical samples suitable for preparing the control plasmid. The LoD was defined as the DNA concentration at which a positive signal was observed in more than 95% of the technical repeats. For 2 copies per reaction, there were no false negative results in any of the 61 repetitions. Thus, the limit of detection for both HAdV-B and HAdV-C was 2 copies per reaction.

### 2.6. Testing on Clinical Samples

**Figure 7:**
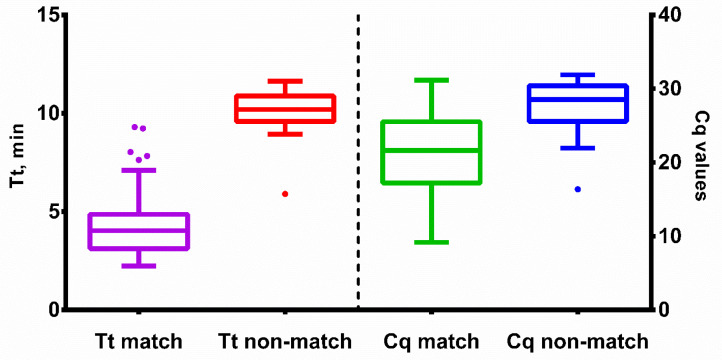
Tt and Cq values of qLAMP and qPCR with clinical samples. Time-to-threshold (Tt) values are presented on the left *Y*-axis, Cq values are plotted against the right *Y*-axis, sample type is shown on the *X*-axis. Match means samples concordant between PCR and qLAMP; non-match means discordant samples. Each sample was analyzed in duplicates; error bars represent one SD; dots represent outliers.

Multiplexed LAMP and PCR assays were validated using nasal swabs taken from 331 patients at G. Speransky Children’s Hospital No. 9 in Moscow, Russia. All study participants signed an informed consent form. The results from qLAMP and PCR assays were in agreement in 299 out of 331 samples (90.3%, Cohen’s coefficient—0.8). For vLAMP and PCR, the results were similar in 300 out of 331 samples (90.6%, Cohen’s coefficient—0.8) (Table S1). Among 31 qLAMP false negative samples, the median Cq value was 28 (27.5 for HAdV-B and 29.25 for HAdV-C). Therefore, we assumed that the primary reason for the relatively low level of agreement between LAMP and qPCR was due to the different limits of detection (LoDs) of these techniques. The LoD for LAMP was lower than that of qPCR. Nevertheless, it was possible to clearly differentiate between matched and unmatched samples based on the Tt and Cq values of qLAMP and qPCR, respectively (Figure 7).

Excluding 23 samples with Cq values higher than 30 (based on our in-house qPCR assay) led to increased concordance: for qLAMP and PCR—93.8 (Cohen’s coefficient—0.87), for vLAMP and PCR—94.2 (Cohen’s coefficient—0.88). It should be noted that the clinical samples were not previously tested using validated methods. We concluded that a high level of agreement between these tests, which target different regions of the HAdVs genome, is sufficient to confirm the presence or absence of HAdV DNA in clinical samples.

## 3. Discussion

Nucleic acid testing (NAT) has become an essential tool in clinical virology, with its high sensitivity, short turnaround time, and ability to detect resistance genes. NAT provides valuable information for early diagnosis and outbreak management. The emergence of PCR has significantly improved the detection of human pathogens, including human adenoviruses. However, this technique requires expensive equipment, which limits its use in resource-constrained settings and makes it unsuitable for point-of-care testing. These factors have led to increasing interest in isothermal amplification techniques like LAMP. In the context of pandemics like COVID-19, LAMP could save valuable resources such as equipment and personnel time. 

In this study, we have developed a multiplex LAMP assay for the detection of known adenoviruses infecting the human respiratory tract and have compared it with our in-house multiplex PCR assay. Being cancatemers of sequences between F3/B3 primers, LAMP products can be distinguished by melting analysis because each specific LAMP amplicon has a specific melting temperature similar to PCR products. Our results suggest that LAMP multiplexing requires rigorous primer selection and several optimization steps to achieve satisfactory results. It is worth noting that the multiplexing of HAdV-B, -C and -E primer sets did not result in a decrease in the sensitivity compared to monoplex reactions. Still, we were unable to completely eliminate non-specific amplification in non-template reactions. These unwanted byproducts limited the duration of LAMP and possibly reduced its sensitivity. Off-target amplification may result from suboptimal primer design, where important parameters are not considered. Currently, there is no open-source software available for multiplex LAMP primer design. Such software could drastically facilitate primer design and, consequently, the analytical characteristics of LAMP assays.

In the context of laboratory diagnostics, the novel qLAMP assay provides the ability to distinguish between different types of HAdV (B, C, and E), using melting curve analysis. This not only allows for viral typing but also allows for the identification of true negative samples based on their respective melting peaks. The qLAMP improves the analytical characteristics of the assay by reducing the probability of false positive and negative results. Even with the addition of melting curve analysis, qLAMP is faster than qPCR, taking only 57 min. LAMP results can also be visualized using intercalating or pH-dependent dyes, making it possible for naked-eye reaction product detection. vLAMP is also suitable for point-of-care testing, with results available in just 35 min compared to 92 min for qPCR. Here, the results of vLAMP were almost identical to qLAMP. However, naked-eye assessment is not always accurate due to the subjectivity associated with the operator’s interpretation, and it also requires careful selection of dye.

We tested the developed LAMP assay on 331 clinical samples. In terms of sensitivity, the triplex LAMP was comparable to in-house PCR, with a concordance rate of 90–94%. However, the LoD of the LAMP assay was 200-fold higher for HAdV-B, -C, and, presumably, for HAdV-E. Despite this, our LAMP results were comparable to those obtained with approved commercially available kits in the Russian Federation, including ARVI-screen-FL (Federal Budget Institute of Science «Central Research Institute of Epidemiology», Moscow, Russia)—5 × 10^3^ genome equivalents per ml, ARVI-complex (DNA-Technology, Moscow, Russia)—4 × 10^3^ copies per µL, AmpliPrime ARVI-complex (NextBio, Moscow, Russia)—1 × 10^3^ copies per µL. Currently, there are no commercially available LAMP kits available for HAdV diagnosis.

It is worth noting that LAMP-based assays have several limitations. Four or six primers make LAMP more specific than PCR but hamper the detection of highly variable pathogens. Viral genomes often accumulate novel mutations quickly, which can weaken the binding of primers, resulting in low amplification efficiency. We cannot precisely predict the impact of these hypothetical mismatches between the primer and template on the amplification results. Therefore, including additional target regions in the LAMP assay, such as through multiplexing, could prevent possible false-negative results. However, simultaneously amplifying several targets in LAMP is a challenging task, which drastically limits the number of regions and pathogens that can be analyzed in a single tube. Another limitation of qLAMP is the need for a real-time PCR machine similar to qPCR. Thus, qLAMP can only be performed in specialized diagnostic laboratories with relatively advanced equipment. Nevertheless, HAdV typing is available by using simple vLAMP and three separate reactions with specific primer sets.

We assume that the novel LAMP assay can detect all known respiratory human adenoviruses. However, this statement is based solely on in silico analyses with alignments of HAdV sequences available in the NCBI database. Due to the lack of positive subtyped clinical samples, we have not conducted such in vitro experiments. Under the assumption that the severity of adenoviral diseases correlates with the measured Cq values [43,44], and that a low viral load indicates less contagiousness [45,46], we conclude that the developed LAMP assay has potential for clinical use.

## 4. Materials and Methods

### 4.1. Design of LAMP Primers

The National Center for Biotechnology Information (NCBI) Nucleotide database was used and aligned using the Multiple Sequence Alignment by Fast Fourier Transform (MAFFT) method. Several conserved regions were identified as candidate regions for primer design:

1. HAdV-B, control protein E1A, region 33226–33443 (NC_011202.1, https://www.ncbi.nlm.nih.gov/nuccore/NC_011202.1/, accessed on 2 January 2022);
2. HAdV-C, control protein E1B, region 3300–3489 (NC_001405.1, https://www.ncbi.nlm.nih.gov/nuccore/NC_001405/, accessed on 2 January 2022);
3. HAdV-E, single-stranded DNA-binding protein E2A, region 21996–22186 (NC_003266.2, https://www.ncbi.nlm.nih.gov/nuccore/NC_003266.2/, accessed on 2 January 2022);

Primers were designed using the Primer Explorer version 5 software (Eiken Chemical Co., Tokyo, Japan) and the NEB LAMP Primer Design Tool, version 1.3.0 (New England Biolabs, Inc., Ipswich, MA, USA). A set of six primers (F3, B3, FIP, BIP, LF, and LB) were selected for each of the three HAdV species: HAdV-B, HAdV-C and HAdV-E (Table 1).

All primers were tested for their specificity and sensitivity to different serotypes of HAdV by aligning them against extracted sequences and using a BLAST search against the GenBank database. If mismatches were found more than 5 nucleotides away from the 3′ end of F3 or B3, or the 3′ and 5′ ends of F1c or B1c, and LF or LB, the primers were deemed suitable.

### 4.2. Design of PCR Primers

Primers were designed using the same alignments as the LAMP primers using the Primer3 software (https://github.com/primer3-org/primer3, accessed on 31 January 2022). Highly conserved regions were identified using in-house Python scripts. Different conserved genes were selected for the assay:

1. HAdV-B—L3 gene, region 26361–26434 (NC_011202.1, https://www.ncbi.nlm.nih.gov/nuccore/NC_011202.1/, accessed on 2 January 2022);
2. HAdV-C—capsid protein precursor pVI, region 25927–25996 (NC_001405.1, https://www.ncbi.nlm.nih.gov/nuccore/NC_001405/, accessed on 2 January 2022);
3. HAdV-E—E3 gene, region 27467–27544 (NC_003266.2, https://www.ncbi.nlm.nih.gov/nuccore/NC_003266.2/, accessed on 2 January 2022).

The length of the amplicons was minimized for each HAdV type (HAdV-B—74 bp, HAdV-C—70 bp, HAdV-E—78 bp). The annealing temperature of the primers was similar to 62 °C, while the probes were 4–5 °C higher. For each region, two primers and one probe were designed for each sample. Additionally, a pair of primers that target the human ALB gene (NC_000004.12) was added as an internal control to the multiplex (Table 2).

Primer specificity was tested using the BLAST algorithm to ensure that all the oligonucleotides mapped only to the corresponding HAdV genome. An analysis of potential primer dimers was carried out using OligoAnalyzer 1.0.2 (by Teemu Kuulasmaa).

### 4.3. Clinical and Standard Samples

This study was approved by the Ethics Committee of the ICBFM SB RAS, Protocol No. 2 dated 21 April 2020.

Clinical samples (nasal swabs) were collected from patients at the Speransky Children’s Hospital #9 in Moscow, Russia. In total, we obtained 221 HAdV-positive and 110 HAdV-negative samples from patients with symptoms of acute respiratory infections. Cq and Tt values can be found in Table S2. To create control samples for LAMP, fragments of genomes of HAdV-B, -C and -E were synthesized and cloned into the pBluescriptSK+ vector by Shanghai RealGene Bio-Tech, Inc. (Shanghai, China), which provided control plasmids pHADVB, pHADVC and pHADVE. The control plasmids were then linearized using the restriction endonuclease BamHI and quantified using digital droplet PCR (ddPCR) with primers HAdVB-F3-1/HAdVB-B3-1, HAdVC-F3-1/HAdVC-B3-1, HAdVE-F3-2/HAdVE-B3-2 (Table 1).

Control plasmids for the PCR assay were prepared in-house using the Quick-TA kit (Evrogen, Russia) in accordance with the manufacturer’s instructions. Amplicons from the PCR with clinical samples that had the highest viral load (based on the results of the PCR) were used for cloning. The structure of the plasmid clones was confirmed by Sanger sequencing, using the Big Dye Terminator kit 3.1 (Applied Biosystems, Waltham, MA, USA) and ABI 3730 genetic analyzer (Applied Biosystems, USA) in the laboratory of antimicrobial drugs at ICBFM SB RAS according to the manufacturer’s protocol. The plasmids were serially diluted within the range of 100–105 in a buffer containing 10 mM Tris-HCl pH 8.0, 2.5 μg/mL yeast RNA, and 0.01% NaN_3_.

### 4.4. Droplet Digital PCR

Plasmid quantification was performed using digital PCR with the QX200™ Droplet Digital™ PCR System (Bio-Rad, Hercules, CA, USA) following the manufacturer’s instructions. The reactions were carried out in a total volume of 20 µL containing the plasmid at approximate concentrations of 1, 10, 100, 10^3^ and 10^4^ copies per reaction. The reaction mixture also included 1× ddPCR EvaGreen Supermix (Bio-Rad, Hercules, CA, USA), 900 nM F3/B3 primers from each of the HAdV set (Table 1) or PCR primers (Table 2), as well as 20 µL of PCR mix and 70 µL droplet generation oil. For droplet formation, the PCR mixture was placed into the corresponding wells of a DG8 cartridge, followed by a 40 μL transfer to a 96-well plate. The plate was sealed with foil and placed in the C100 thermal cycler (Bio-Rad, Hercules, CA, USA). The amplification protocol included 96 °C for 10 min, followed by 45 cycles at 96 °C for 30 s, 58 °C for 60 s, with a final step at 98 °C for 10 min. The ramp rate for all steps was 2 °C/s. The droplets were analyzed using a dedicated droplet reader, and the resulting data were then processed using the QuantaSoft software version 1.0 from Bio-Rad (Hercules, CA, USA).

### 4.5. Quantitative LAMP (qLAMP)

Standard LAMP reactions contained 20 µL of the following components: 1× reaction buffer for Gss-Sto polymerase (40 Mm Tris-HCl pH 8.9, 10 mM (NH_4_)_2_SO_4_, 10 mM KCl, 8 mM MgSO_4_, 2.5% DMSO, 0.1% Triton X100), 1.5 mM each of dNTP, 0.4 µM each of external primer (F3/B3), 0.8 µM loop primers (LF/LB), 1.6 µM internal primers (FIP/BIP) (Table 1), DNA template, 3 units of Gss-Sto-polymerase [47], and 1 µM intercalating dye SYTO-82. The exact amount of each component was optimized and specified in the results section for each experiment. The reactions were carried out in a CFX96 thermocycler (Bio-Rad, Hercules, CA, USA) using the following program: 120 cycles of primer annealing and elongation, each at 60 °C for 20 s with a fluorescence signal registration in the HEX channel; post-amplification melting of amplification products in the range of 75–95 °C. The results of isothermal amplification were evaluated using the Tt values (time-to-threshold), which is the time interval before the point where the amplification curve intersects with a threshold line. The typing of HAdV was based on the melting temperature obtained from a melting curves analysis. Additionally, 8% polyacrylamide gel electrophoresis was conducted in 0.5% TBE buffer to validate the length and the pattern of LAMP products in both templates and no-template controls.

### 4.6. Quantitative PCR (qPCR)

PCR reactions (20 µL) contained 1× PCR buffer (64 mM Tris-HCl pH 8.9, 16 mM (NH_4_)_2_SO_4_, 0.05% Tween 20, 3 mM MgCl_2_, 0.002% NaN_3_), 0.6 µM primers (forward and reverse from PCR assay) and 0.15 µM probes (Table 2), 2 units of Taq-polymerase (SibEnzyme, Novosibirsk, Russia), and a DNA template. The amount of DNA template used in each experiment is specified in the results section. The PCR reactions were carried out in a CFX96 thermocycler (Bio-Rad, Hercules, CA, USA).

The program for PCR primers included the following steps: activation of Taq-polymerase for 15 min at 96 °C, followed by 45 cycles of denaturation at 95 °C for 10 s, annealing and elongation at 60 °C for 40 s with the registration of a fluorescent signal in FAM, ROX and Cy5 channels.

The program for LAMP primers included the following steps: activation of Taq-polymerase for 15 min at 96 °C, followed by 45 cycles of denaturation at 95 °C for 10 s, annealing at 60 °C for 10 s and elongation at 72 °C for 20 s with the registration of a fluorescent signal in a HEX or FAM channel.

### 4.7. Visual LAMP (vLAMP)

Commercially available SYBR Green I dye (Lumiprobe, Moscow, Russia) was utilized for the visual detection of vLAMP results. A mixture of 20% sucrose and 1700-fold SYBR-Green was added to PCR cap strips (1.8 µL per each cap) and dried at 65 °C for 40 min. The caps were used to seal the PCR strips with the LAMP reaction mixtures. Standard vLAMP reactions (20 µL) included the same reagents as qLAMP (see the Quantitative LAMP section); however, SYTO-82 was not included in the mixture. The reactions were incubated for 35 min at 60 °C in a DB-45 thermostat for strips (Biosan SIA, Rīga, Latvia), following which the strips were flipped and shaken to dissolve any SYBR Green pellets on the caps. If the reaction color shifted from orange to green, the sample was considered positive for HAdV when the color of the reaction remained orange, the specimen was marked as negative.

### 4.8. Evaluation of the Limit of Detection, Clinical Sensitivity and Specificity of LAMP and PCR

The limit of detection (LoD) was assessed by varying the concentration of a control plasmid in the reaction mixtures: 1000, 750, 500, 375, 250, 100 or 50 copies per reaction. A total of 31 technical replicates were used for multiplex LAMP, and typing of HAdVs was performed based on a melting temperature analysis. The LoD was defined as the HAdV DNA concentration that provided a relevant melting peak for HAdV amplification products in 95% of the technical replicates.

The analytical specificity of the method was evaluated in silico by using the BLAST algorithm to compare the sequences of HAdV with common human viral and bacterial pathogens such as Haemophilus influenzae, Streptococcus pneumoniae and pyogenes, seasonal coronaviruses and human rhinoviruses.

The clinical sensitivity and specificity of the test were evaluated using samples from patients at Speransky Children’s hospital No. 9 in Moscow, Russia. The DNA was isolated from the samples using the RIBO-prep kit (AmpliSens, Moscow, Russia) following the manufacturer’s protocol. The extracted DNA was then tested using quantitative PCR and a multiplex LAMP assay.

### 4.9. Results Validation

Sanger sequencing was performed to analyze clinical samples that were negative in the LAMP assay, but positive in the PCR test, when the DNA concentration in the sample was higher than the LoD of the LAMP assay or when mistyping by two test-systems was detected. PCR was performed using F3/B3 primers from the LAMP sets or forward and reverse primers of PCR. The resulting products were sequenced using the Big Dye Terminator kit 3.1 (Applied Biosystems) and ABI 3730 genetic analyzer (Applied Biosystems, USA) at the Laboratory of antimicrobial drugs of ICBFM SB RAS according to the manufacturer’s protocol. Raw sequencing data were analyzed using the Chromas software, version 2.6.4 (Technelysium Pty Ltd., Queensland, Australia) and mapped to the HAdV reference genomes using the MAFFT algorithm in Unipro UGENE software, version 45.1 [48] or analyzed using the BLAST algorithm.

## 5. Conclusions

The colorimetric LAMP and real-time LAMP assays developed in this study allow for the specific and sensitive identification of adenoviruses that infect the human respiratory tract. The real-time variant of LAMP can also be used to differentiate between different types of adenoviruses. Colorimetric LAMP, on the other hand, does not require specialized equipment or complex procedures and therefore offers a more accessible alternative. Both methods have their own unique advantages and analytical strengths and are comparable to commercially available PCR kits. They could potentially be integrated into point-of-care diagnostic testing protocols to enhance early detection and treatment of adenovirus infections.

## Figures and Tables

**Table 1:** Primers for multiplex LAMP assay.

Target	LAMP Primer	Sequence 3′–5′
HAdV-B	F3	AGAGACGGAAGAATCATGTT
	B3	AGAAAATGCTCCTTCTTTTGG
	LF	GCGATAGATGCCATCTGC
	LB	GCTTCCAACAAAGCCTCC
	FIP	CTTTTTCACCAACACAGTGGGGGCAGTACTTCAAATTGTAGATCG
	BIP	TCAAAAGAAATGCGATTTTCAAGGTCTTTTGTTTTTGGATGTGCG
HAdV-C	F3	GGGGTGTTTGACATGACCAT
	B3	CATCGCTAGAGCCAAACTCA
	LF	TCTGCACCTGGTGCGGGTC
	LB	GGATGTGACCGAGGAGCTGAGG
	FIP	GTTTACCGCCACACTCGCAGGGATCTGGAAGGTGCTGAGGT
	BIP	TAGGAACCAGCCTGTGATGCTGCCAGCACCAAGTGATCGG
HAdV-E	F3	GCGAGAGTTGCGGTACAC
	B3	GCGATTGCAACCACAAGC
	LF	TGGCGAGCGTGAAGCATC
	LB	GCGTTGGCCATCCCAAAGG
	FIP	ATCACCGACGCGACGGTGCGGGGTTGCAGCACTGG
	BIP	CCGTCCACGTCGAGGTCTTCGCTGCGTGCCCACCATG

**Table 2:** Primers for multiplex PCR assay.

Target	Primer	Sequence 3′–5′
HAdV-B	F	GTATCACAAGAGCGAAGACCAA
	R	GCGCGCAGTACTTGTTGAAGAG
	P	R6G-CAGCGCACTCTCGAGGACGCCG-BHQ1
HAdV-C	F	CTCCGGGGTTGAAACTCACT
	R	TAGTCCTCAGGTACAAATTTGCG
	P	ROX-AGGTAAGCCGACGTCCACAGCCCC-BHQ2
HAdV-E	F	CGCCGTTGTTAACCACTGCTAC
	R	CTTCTGCGGGTGGAAAAAGTAAG
	P	FAM-CGGAGTCCTGCTAAACGGTCCCG-BHQ1
ALB	F	GACTTGCCAAGACATATGAAACC
	R	TCCAACAATAAACCTACCACTTTG
	P	Cy5.5-TGCTGTGCCGCTGCAGATCC-BHQ3

## Data Availability

The raw data supporting the conclusions of this article will be made available by the authors on request.

## Supplementary Materials

The supporting information can be downloaded at: https://www.mdpi.com/article/10.3390/ijms25137215/s1.
