# Supplementary material for: A Novel Multiplex LAMP Assay for the Detection of Respiratory Human Adenoviruses
Source: Int J Mol Sci. 2024 Jun 29;25(13):7215. doi: 10.3390/ijms25137215 (PMC11241107; doi:10.3390/ijms25137215)
Supplement: Supplementary file 1 [file ijms-25-07215-s001.zip › ijms-3042783-supplementary.pdf]

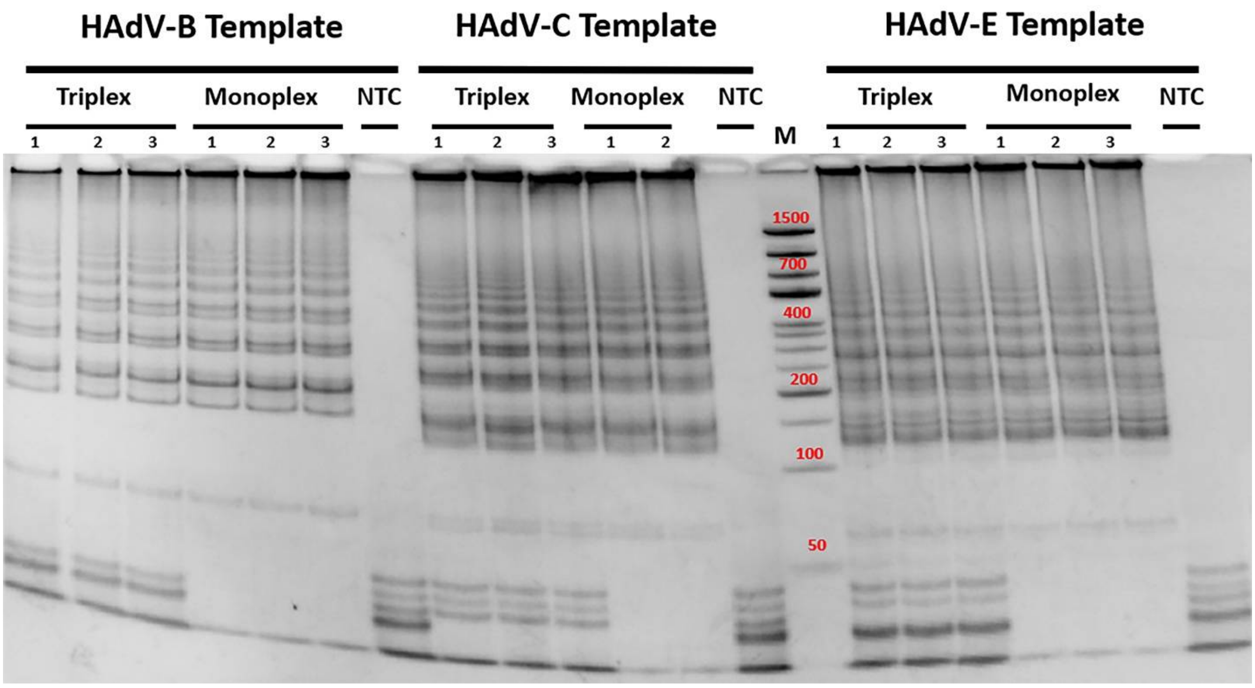

Figure S1. LAMP products for each HAdV type. Each HAdV type-specific DNA controls were assesed in triplex (B, C, and E) and monoplex (type-specific primer mixture) reactions. M — DNA marker, sizes of bands are specified by red; NTC — no template controls.

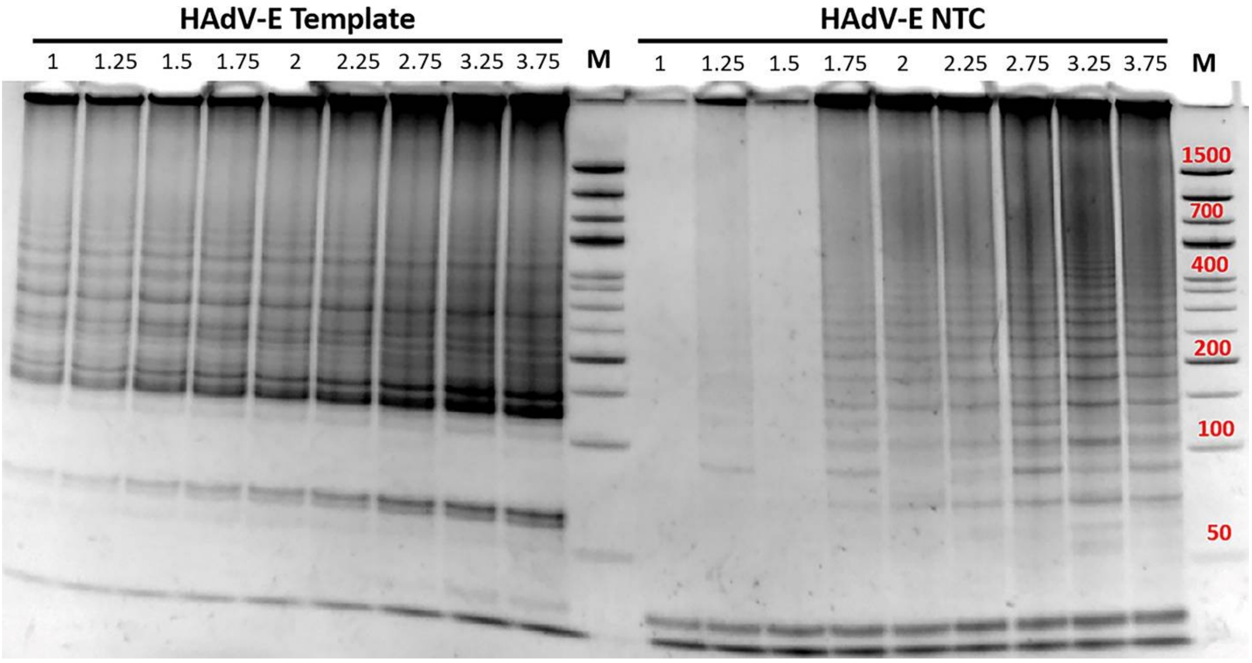

Figure S2. HAdV-E primers titration in monoplex LAMP. Template concentration was 2×10^{3} copies. Primer concentration was titrated in the range of 1–3.75×. 1× concentration was 1.6 μM FIP/BIP, 0.8 μM LF/LB, 0.4 μM F3/B3 primers. M — DNA marker, sizes of bands are specified by red; NTC — no template controls.

Table S1. Comparison of the results of LAMP and PCR on clinical samples.

		**All samples**			
		qLAMP		vLAMP	
		Positive	Negative	Positive	Negative
qPCR	Positive	190	31	191	30
	Negative	1	109	1	109

		**Samples with Cq values below 30**			
		qLAMP		vLAMP	
		Positive	Negative	Positive	Negative
qPCR	Positive	180	18	181	17
	Negative	1	109	1	109

Table S2. Clinical samples testing results.

Sample	PCR, Cq	qLAMP, min	qLAMP, Tm	vLAMP results	*Human adenovirus* species
1	20.0	9.1	85.0	Positive	B
2	17.7	8.3	85.0	Positive	B
3	27.6	30.7	None	Negative	B
4	19.3	12.5	89.0	Positive	C
5	29.0	15.3	89.0	Positive	C
6	31.9	29.3	93.0	Negative	B
7	30.1	32.6	94.5	Negative	B
8	30.3	30.6	93.0	Negative	B
9	31.2	27.9	88.5	Positive	C
10	14.3	8.7	85.0	Positive	B
11	17.5	9.8	85.0	Positive	B
12	16.4	8.3	85.5	Positive	B
13	12.5	6.7	85.0	Positive	B
14	18.4	10.8	88.5	Positive	C
15	25.6	18.5	88.5	Positive	C
16	18.3	12.8	88.5	Positive	C
17	30.7	29.3	None	Positive	C
18	27.2	16.4	89.0	Positive	C
19	18.8	9.0	85.0	Positive	B
20	23.4	9.2	85.0	Positive	B
21	28.1	16.3	85.5	Positive	B
22	26.9	19.7	85.0	Negative	B
23	23.8	9.9	85.0	Positive	B
24	21.6	12.9	89.0	Positive	C
25	28.7	14.2	85.0	Positive	B
26	15.3	10.4	88.5	Positive	C
27	27.4	28.8	88.5	Positive	C
28	17.2	10.2	88.5	Positive	C
29	27.5	15.0	88.5	Positive	C
30	22.4	11.9	85.5	Positive	B
31	20.8	12.5	85.5	Positive	B
32	16.4	34.1	None	Negative	B
33	24.2	26.8	None	Negative	B
34	22.2	14.4	88.5	Positive	C
35	28.1	11.8	85.0	Positive	B

Sample	PCR, Cq	qLAMP, min	qLAMP, Tm	vLAMP results	*Human adenovirus* species
36	14.1	6.7	85.0	Positive	B
37	9.2	7.3	85.0	Positive	B
38	27.4	14.4	85.5	Negative	B
39	26.7	13.7	85.5	Positive	B
40	21.5	9.9	85.0	Positive	B
41	21.7	13.4	88.5	Positive	C
42	19.6	13.0	89.0	Positive	C
43	17.6	8.8	85.0	Positive	B
44	22.1	12.9	89.0	Positive	C
45	22.2	15.3	88.5	Positive	C
46	30.3	30.2	89.0	Negative	C
47	18.2	12.0	88.5	Positive	C
48	25.6	34.5	None	Negative	B
49	16.8	8.4	85.0	Positive	B
50	14.3	7.9	85.0	Positive	B
51	28.1	31.7	None	Negative	C
52	18.5	11.4	85.0	Positive	B
53	28.6	31.9	90.5	Negative	B
54	31.8	20.2	88.5	Positive	B
55	20.3	9.9	88.5	Positive	C
56	17.3	12.9	89.0	Positive	C
57	19.4	14.2	88.5	Positive	C
58	15.8	8.2	85.0	Positive	B
59	14.4	11.8	88.5	Positive	C
60	30.7	13.2	85.0	Positive	B
61	25.1	28.2	89.0	Positive	C
62	27.3	12.1	85.0	Positive	B
63	24.7	16.9	88.5	Positive	C
64	22.2	12.8	88.5	Positive	C
65	27.6	16.1	85.0	Positive	B
66	13.5	7.4	85.0	Positive	B
67	21.0	12.1	88.5	Positive	C
68	28.2	23.5	88.5	Positive	C
69	22.0	29.7	91.0	Negative	B
70	16.2	15.7	88.5	Positive	C
71	31.8	28.1	92.5	Negative	C
72	24.6	18.1	89.0	Positive	C
73	24.7	17.8	89.0	Positive	C
74	27.9	18.8	85.0	Positive	C
75	22.9	21.9	88.5	Positive	C
76	27.1	18.2	89.5	Positive	C
77	23.1	16.6	85.0	Negative	B
78	16.9	12.7	89.0	Positive	C
79	22.6	10.5	85.0	Positive	B
80	23.8	12.7	85.5	Positive	B
81	16.0	12.6	89.0	Positive	C
82	28.9	31.2	None	Negative	C
83	15.9	10.0	86.0	Positive	B
84	16.1	12.2	88.5	Positive	C
85	27.7	22.9	89.0	Positive	C
86	22.0	11.2	85.5	Positive	B
87	27.4	18.4	89.0	Positive	C

Sample	PCR, Cq	qLAMP, min	qLAMP, Tm	vLAMP results	*Human adenovirus* species
88	26.6	27.7	88.5	Positive	C
89	24.6	16.3	89.0	Positive	C
90	16.7	8.8	85.0	Positive	B
91	18.9	9.1	85.0	Positive	B
92	23.2	21.3	88.5	Positive	C
93	29.1	24.1	88.5	Positive	C
94	26.4	27.8	92.5	Positive	B
95	30.1	28.2	92.0	Negative	B
96	30.5	20.5	88.5	Positive	B
97	18.3	9.1	88.0	Positive	C
98	16.7	9.3	88.0	Positive	C
99	16.1	10.2	88.5	Positive	C
100	17.5	8.1	85.0	Positive	B
101	24.4	11.5	84.5	Positive	B
102	17.6	7.1	84.5	Positive	B
103	28.5	27.7	88.5	Positive	C
104	11.3	7.4	88.5	Positive	C
105	18.4	9.4	88.0	Positive	C
106	19.8	10.0	88.5	Positive	C
107	11.5	8.0	88.5	Positive	C
108	20.6	13.6	88.5	Positive	C
109	26.3	7.5	88.5	Positive	C
110	25.4	11.7	84.5	Positive	B
111	25.6	12.0	88.0	Positive	C
112	27.5	11.8	88.0	Positive	C
113	27.9	14.8	88.0	Positive	C
114	22.6	12.7	88.5	Positive	C
115	23.8	13.0	88.0	Positive	C
116	14.0	10.5	88.5	Positive	C
117	24.1	12.1	88.5	Positive	C
118	25.5	N/A	None	Negative	B
119	31.9	20.0	88.5	Positive	C
120	19.2	8.7	85.0	Positive	B
121	31.5	31.5	None	Negative	B
122	29.7	17.0	88.5	Positive	C
123	21.9	13.0	89.0	Positive	C
124	30.0	33.1	None	Positive	B
125	23.6	15.9	88.5	Positive	C
126	23.2	10.0	85.0	Positive	B
127	14.7	11.1	88.0	Positive	C
128	23.5	11.5	85.0	Positive	B
129	22.4	9.3	85.0	Positive	B
130	16.5	9.2	85.0	Positive	B
131	20.0	12.0	85.0	Positive	B
132	13.4	7.7	85.0	Positive	B
133	19.5	9.1	85.0	Positive	B
134	18.6	9.1	85.0	Positive	B
135	11.6	7.4	85.0	Positive	B
136	30.5	20.5	88.5	Positive	C
137	24.9	11.5	85.0	Positive	B
138	24.1	15.6	85.5	Positive	B
139	24.7	9.8	85.0	Positive	B

Sample	PCR, Cq	qLAMP, min	qLAMP, Tm	vLAMP results	*Human adenovirus* species
140	15.8	9.3	85.0	Positive	B
141	14.6	9.0	85.0	Positive	B
142	23.3	12.8	88.5	Positive	C
143	29.1	16.7	88.5	Positive	C
144	8.9	7.8	85.0	Positive	B
145	27.1	33.6	None	Positive	B
146	28.9	12.2	85.0	Positive	B
147	31.8	31.6	None	Positive	B
148	23.7	10.7	85.0	Positive	B
149	23.0	25.0	89.0	Positive	C
150	15.8	10.2	85.5	Positive	B
151	27.7	23.5	88.5	Positive	C
152	22.2	10.2	84.5	Positive	B
153	26.4	36.7	None	Negative	B
154	17.3	9.2	84.5	Positive	B
155	20.5	13.5	89.0	Positive	C
156	23.8	14.9	88.5	Positive	C
157	21.2	15.0	88.5	Positive	C
158	22.2	12.0	88.5	Positive	C
159	12.6	8.2	85.0	Positive	B
160	17.1	7.9	85.0	Positive	B
161	20.4	12.1	88.5	Positive	C
162	27.2	32.2	None	Positive	B
163	27.5	16.8	88.5	Positive	C
164	22.2	9.7	85.0	Positive	B
165	18.9	12.9	88.5	Positive	C
166	18.0	31.7	89.5	Positive	C
167	32.1	22.6	85.0	Positive	B
168	24.8	16.0	88.5	Positive	C
169	16.2	7.4	85.0	Positive	B
170	20.4	16.6	89.5	Positive	C
171	24.3	9.9	84.5	Positive	B
172	17.1	10.3	88.5	Positive	C
173	26.7	15.0	88.5	Positive	C
174	31.4	35.6	None	Negative	C
175	19.7	12.2	88.0	Positive	C
176	19.2	11.8	88.5	Positive	C
177	27.1	18.6	84.5	Positive	B
178	27.1	30.7	88.5	Positive	C
179	13.3	7.8	85.0	Positive	B
180	33.5	32.8	None	Negative	B
181	22.6	37.8	None	Negative	B
182	31.8	29.8	None	Negative	C
183	19.3	11.2	84.5	Positive	B
184	18.7	9.1	85.0	Positive	B
185	22.0	12.4	85.0	Positive	B
186	29.2	22.5	89.0	Positive	C
187	26.0	31.1	None	Positive	B
188	23.1	16.0	85.0	Negative	B
189	22.4	10.0	84.5	Positive	B
190	29.7	15.3	88.5	Positive	C
191	15.4	7.4	85.0	Positive	B

Sample	PCR, Cq	qLAMP, min	qLAMP, Tm	vLAMP results	*Human adenovirus* species
192	21.3	33.3	None	Positive	C
193	25.3	15.1	88.5	Positive	C
194	19.7	10.4	85.0	Positive	B
195	28.3	33.5	None	Negative	C
196	18.1	9.4	85.0	Positive	B
197	30.3	15.1	88.5	Positive	C
198	15.4	9.1	85.0	Positive	B
199	30.4	21.3	88.5	Negative	C
200	6.5	7.7	88.0	Positive	C
201	15.8	10.6	88.5	Positive	C
202	15.2	16.1	85.0	Positive	B
203	24.3	12.7	85.0	Positive	B
204	26.8	21.9	88.5	Positive	C
205	25.7	17.5	89.0	Positive	C
206	22.0	9.4	84.5	Positive	B
207	21.5	12.7	88.0	Positive	C
208	17.9	10.6	88.5	Positive	C
209	26.3	16.0	88.5	Positive	C
210	28.2	16.8	85.0	Positive	B
211	27.1	10.5	84.5	Positive	B
212	30.6	33.7	None	Negative	B
213	27.4	16.6	88.5	Positive	C
214	29.6	24.7	85.0	Negative	B
215	18.3	10.9	88.0	Positive	C
216	17.1	10.9	88.5	Positive	C
217	17.5	7.1	85.0	Positive	B
218	25.5	9.9	85.5	Positive	B
219	16.3	7.1	85.0	Positive	B
220	31.0	27.7	None	Negative	C
221	18.4	8.1	85.0	Positive	B
222	N/A	N/A	N/A	Negative	-
223	N/A	N/A	N/A	Negative	-
224	N/A	N/A	N/A	Negative	-
225	N/A	N/A	N/A	Negative	-
226	N/A	N/A	N/A	Negative	-
227	N/A	N/A	N/A	Negative	-
228	N/A	N/A	N/A	Negative	-
229	N/A	N/A	N/A	Negative	-
230	N/A	N/A	N/A	Negative	-
231	N/A	N/A	N/A	Negative	-
232	N/A	N/A	N/A	Negative	-
233	N/A	N/A	N/A	Negative	-
234	N/A	N/A	N/A	Negative	-
235	N/A	N/A	N/A	Negative	-
236	N/A	N/A	N/A	Negative	-
237	N/A	N/A	N/A	Negative	-
238	N/A	N/A	N/A	Negative	-
239	N/A	N/A	N/A	Negative	-
240	N/A	N/A	N/A	Negative	-
241	N/A	N/A	N/A	Negative	-
242	N/A	N/A	N/A	Negative	-
243	N/A	N/A	N/A	Negative	-

Sample	PCR, Cq	qLAMP, min	qLAMP, Tm	vLAMP results	*Human adenovirus* species
244	N/A	N/A	N/A	Negative	-
245	N/A	N/A	N/A	Negative	-
246	N/A	N/A	N/A	Negative	-
247	N/A	N/A	N/A	Negative	-
248	N/A	N/A	N/A	Negative	-
249	N/A	N/A	N/A	Negative	-
250	N/A	N/A	N/A	Negative	-
251	N/A	N/A	N/A	Negative	-
252	N/A	N/A	N/A	Negative	-
253	N/A	N/A	N/A	Negative	-
254	N/A	N/A	N/A	Negative	-
255	N/A	N/A	N/A	Negative	-
256	N/A	N/A	N/A	Negative	-
257	N/A	N/A	N/A	Negative	-
258	N/A	N/A	N/A	Negative	-
259	N/A	N/A	N/A	Negative	-
260	N/A	N/A	N/A	Negative	-
261	N/A	N/A	N/A	Negative	-
262	N/A	N/A	N/A	Negative	-
263	N/A	N/A	N/A	Negative	-
264	N/A	N/A	N/A	Negative	-
265	N/A	N/A	N/A	Negative	-
266	N/A	N/A	N/A	Negative	-
267	N/A	N/A	N/A	Negative	-
268	N/A	N/A	N/A	Negative	-
269	N/A	N/A	N/A	Negative	-
270	N/A	N/A	N/A	Negative	-
271	N/A	N/A	N/A	Negative	-
272	N/A	N/A	N/A	Negative	-
273	N/A	N/A	N/A	Negative	-
274	N/A	N/A	N/A	Negative	-
275	N/A	N/A	N/A	Negative	-
276	N/A	N/A	N/A	Negative	-
277	N/A	17.8	85.0	Positive	B
278	N/A	N/A	N/A	Negative	-
279	N/A	N/A	N/A	Negative	-
280	N/A	N/A	N/A	Negative	-
281	N/A	N/A	N/A	Negative	-
282	N/A	N/A	N/A	Negative	-
283	N/A	N/A	N/A	Negative	-
284	N/A	N/A	N/A	Negative	-
285	N/A	N/A	N/A	Negative	-
286	N/A	N/A	N/A	Negative	-
287	N/A	N/A	N/A	Negative	-
288	N/A	N/A	N/A	Negative	-
289	N/A	N/A	N/A	Negative	-
290	N/A	N/A	N/A	Negative	-
291	N/A	22.6	91.5	Negative	-
292	N/A	N/A	N/A	Negative	-
293	N/A	N/A	N/A	Negative	-
294	N/A	N/A	N/A	Negative	-
295	N/A	N/A	N/A	Negative	-

Sample	PCR, Cq	qLAMP, min	qLAMP, Tm	vLAMP results	*Human adenovirus* species
296	N/A	N/A	N/A	Negative	-
297	N/A	N/A	N/A	Negative	-
298	N/A	29.9	92.5	Negative	-
299	N/A	N/A	N/A	Negative	-
300	N/A	N/A	N/A	Negative	-
301	N/A	N/A	N/A	Negative	-
302	N/A	N/A	N/A	Negative	-
303	N/A	N/A	N/A	Negative	-
304	N/A	N/A	N/A	Negative	-
305	N/A	N/A	N/A	Negative	-
306	N/A	N/A	N/A	Negative	-
307	N/A	N/A	N/A	Negative	-
308	N/A	N/A	N/A	Negative	-
309	N/A	N/A	N/A	Negative	-
310	N/A	N/A	N/A	Negative	-
311	N/A	N/A	N/A	Negative	-
312	N/A	N/A	N/A	Negative	-
313	N/A	N/A	N/A	Negative	-
314	N/A	N/A	N/A	Negative	-
315	N/A	N/A	N/A	Negative	-
316	N/A	N/A	N/A	Negative	-
317	N/A	N/A	N/A	Negative	-
318	N/A	N/A	N/A	Negative	-
319	N/A	N/A	N/A	Negative	-
320	N/A	N/A	N/A	Negative	-
321	N/A	N/A	N/A	Negative	-
322	N/A	N/A	N/A	Negative	-
323	N/A	N/A	N/A	Negative	-
324	N/A	N/A	N/A	Negative	-
325	N/A	N/A	N/A	Negative	-
326	N/A	N/A	N/A	Negative	-
327	N/A	N/A	N/A	Negative	-
328	N/A	N/A	N/A	Negative	-
329	N/A	N/A	N/A	Negative	-
330	N/A	N/A	N/A	Negative	-
331	N/A	N/A	N/A	Negative	-
